# Editorial: Exploring the Potential of PSMA-PET Imaging on Personalized Prostate Cancer Treatment

**DOI:** 10.3389/fonc.2022.832747

**Published:** 2022-02-02

**Authors:** Harun Ilhan, Trevor Royce, Xuefeng Qiu, Constantinos Zamboglou

**Affiliations:** ^1^ Department of Nuclear Medicine, University Hospital, Ludwigs-Maximilian Universitaet (LMU) Munich, Munich, Germany; ^2^ Department of Radiation Oncology, University of North Carolina at Chapel Hill School of Medicine, Chapel Hill, NC, United States; ^3^ Flatiron Health, New York, NY, United States; ^4^ Department of Urology, Affiliated Drum Tower Hospital, Medical School of Nanjing University, Nanjing, China; ^5^ Department of Radiation Oncology, Medical Center – University of Freiburg, Faculty of Medicine, University of Freiburg, Freiburg, Germany; ^6^ German Oncology Center, European University Cyprus, Limassol, Cyprus

**Keywords:** prostate specific membrane antigen (PSMA), prostate cancer, personalized and precision medicine (PPM), surgery, radiotherapy

## Introduction

Prostate cancer (PCa) is the second most frequent cancer diagnosis made in men worldwide (1). Accurate and reliable diagnostic medical imaging is a frequent prerequisite for personalized treatment approaches in patients with PCa by enabling, in part, (i) understanding extent of disease, (ii) accurate segmentation of PCa lesions and, (iii) non-invasive tumor characterization, for example, using radiomics or artificial intelligence tools (2).

Prostate Specific Membrane Antigen (PSMA) has been found to be selectively overexpressed in PCa cells (3) and can be traced by radio-labelled peptide ligands in positron emission tomography (PSMA-PET). First studies suggested excellent diagnostic accuracy and a major impact on therapeutic approaches for PSMA-PET in newly diagnosed (4), recurrent (5) or metastatic PCa patients (4). The goal of this Research Topic was to concentrate scientific contributions on the growing evidence of integrating PSMA-PET imaging in personalized PCa treatment concepts.

The Research Topic accepted 15 articles including a total of 126 authors, demonstrating the growing interest in the field of PSMA-PET imaging. The manuscripts of the Research Topic can be divided into the following topics.

## PSMA-PET for Primary Localized PCa

The accurate segmentation of the intraprostatic tumor mass is a prerequisite for precise targeted-biopsy and focal therapy (FT) approaches in patients with localized PCa. The current imaging gold-standard for intraprostatic tumor detection and delineation is multiparametric magnetic resonance imaging (mpMRI) (6, 7). However, mpMRI was reported to be associated with underestimation of the true intraprostatic PCa extent (Kramer et al.). Recently, it has been suggested that PSMA-PET might give complementary information for intraprostatic tumor detection (8) and guidance of FT (9). In this Research Topic, Spohn et al. compared manual and semi-automatic methods for intraprostatic tumor delineation based on 18F-PSMA-1007 PET/CT images. By using whole-section surgery specimen as the standard of reference the authors proposed several methods with high sensitivity or high specificity. In another work by Spohn et al. the authors used the same methodology in terms of histology reference to perform an *in-silico* radiotherapy planning study (The authors simulated a focal radiotherapy dose escalation based on PSMA-PET and mpMR images and demonstrated that a dosimetric sparing of the intraprostatic urethra might increase the therapeutic ratio.

## PSMA-PET for Recurrent PCa

Biochemical recurrence (BCR) after primary curative intent radiotherapy or radical prostatectomy represents one of the major challenges in the management of PCa. In the recent years, multiple ^68^Ga- and ^18^F-labelled PSMA-targeting radiotracers have been introduced and recommended in several guidelines (10–12). Furthermore, ^68^Ga-PSMA-11 and ^18^F-DCFPyL received recent FDA-approval for imaging of BCR (13, 14).

This Research Topic includes two meta-analyses evaluating the value of several PET-radiopharmaceuticals for the detection of BCR. Wang et al. included 46 studies and compared the three ^18^F-labeled radiotracers ^18^F-choline targeting the phospholipid metabolism, the amino acid ^8^F-Fluciclovine, and ^18^F-labelled PSMA-targeting tracers including PSMA-1007, rhPSMA-7, and DCFPyL. Highest detection rates, even at low PSA levels were observed for ^18^F-PSMA tracers, with a sensitivity of 58% at PSA levels of <0.5 ng/ml compared to 35% and 23% for ^18^F-Choline and ^18^F-Fluciclovine, respectively. In a detailed review and meta-analysis on detection rates for ^18^F-DCFPyL, Sun et al. included 844 patients from 9 studies. With a pooled sensitivity of 88.8% at PSA levels ≥0.5 ng/ml and 47.2% at <0.5 ng/ml, ^18^F-DCFPyL provides high detection rates for BCR despite high heterogeneity in the overall cohort.

The impact of PSMA-PET imaging on the therapeutic management of PCa patients represents another major aspect of this Research Topic. In a multicenter retrospective analysis, Schmidt-Hegemann et al. evaluated biochemical recurrence-free survival (BRFS) after salvage radiotherapy (RT) in a cohort of 459 patients without lymph node or distant metastasis determined by conventional imaging and additional ^68^Ga-PSMA-PET or conventional imaging alone. The authors did not find any significant impact of prior ^68^Ga-PSMA PET on BRFS despite more adverse clinical features in the PET cohort. These results indicate that salvage RT should not be postponed until a PSMA PET-positive result is observed in patients with BCR. Several work-groups all over the globe have evaluated the impact of different PSMA-PET tracers on salvage RT. The high sensitivity of PSMA-PET, specifically for small lymph node and bone metastases has a high impact on target volume definition. According the article by Bottke et al. including only patients with PSA levels ≤0.5 ng/ml, PSMA-PET has a major impact on the target volume definition in 17% and a minor impact in 11%. According to the authors, PSMA-PET based RT might have impact on patients survival. Vogel et al. evaluate the toxicity and outcome of dose escalated salvage RT (DE-SRT) after PSMA-PET compared to conventional salvage RT with. There were no significant differences regarding toxicity rates and the majority of patients should PSA response indicating the feasibility of DE-SRT for personalized RT planning.

Finally, this Research Topic also includes an article on the administrative challenges when imaging PCa patients with BCR. Young et al. provide a detailed description of a PSMA-PET registry in Ontario, Canada including the impact of PSMA-PET imaging on patient management, stakeholder perspectives and interviews. They provide data for ^18^F-DCFPyL in more than 1700 men since 2018. The main idea is to summarize important real world data to provide improved access to novel PET radiopharmaceuticals also in the future.

## PSMA-PET for Metastatic PCa

Local therapy applications in the metastatic setting is one of the exciting developing frontiers of prostate cancer treatment; for example the randomized STOMP trial (15) which found an androgen deprivation therapy-free survival benefit with metastasis-directed therapy (e.g., ablation with stereotactic radiotherapy) for oligorecurrent prostate cancer. The improved performance of PSMA-based imaging techniques amplify this excitement, with the potential to detect earlier metastases. Henkenberens et al. add to the developing body of literature in this area by reporting their experience of 42 patients with oligometastatic CRPC (141 PSMA positive metastases) receiving radiation to all PSMA positive lesions. Their results further suggest such approaches may delay the need for systemic therapies (eg second-line systemic treatment free survival).

Beyond target delineation for the above local therapy applications, PSMA imaging will also likely be valuable in assessing systemic treatment response for metastatic disease. In this context it should be mentioned that PSMA theranostics may result in damage to some PSMA expressing normal tissues such as salivary glands during PSMA-radioligand therapy. Mittlmeier et al. put effort into characterizing and standardizing PSMA-measured metastatic lymph node treatment responses by correlating PSMA-based tumor volumes with a CT reference in fifty patients with metastatic prostate cancer. In their investigation, they derive a proposed SUV threshold value for this purpose. These sorts of investigations will lay the groundwork for future clinical research as PSMA-applications continue to expand. Importantly, these applications can enhance imaging performance by appropriately accounting for normal tissues (ie physiologic uptake of radiotracer). In this Research Topic, Shi et al. published their efforts into characterizing this as it relates to peripheral ganglia physiologic uptake versus pathologic lymph node metastases uptake among 138 prostate cancer patients who underwent both PSMA and FDG scans (Shi et al.; Shi et al.).

## Conclusion

The evolving field of PSMA-targeted diagnostic imaging and therapeutics (theranostics) promise to advance the management of PCa patients in all stages of the disease. Exciting opportunities abound with PSMA-theranostics currently in the discovery pipeline. In a Mini Review by Ng et al. a vision for multidisciplinary use of PSMA theranostics was presented. We fully agree with Ng et al., who conclude by highlighting that the collaboration across the multidisciplinary prostate cancer team will be essential in maximizing the impact of these novel techniques.

## Author Contributions

All authors contributed to the article and approved the submitted version.

## Conflict of Interest

Authors TR was employed by Flatiron Health.

The remaining authors declare that the research was conducted in the absence of any commercial or financial relationships that could be construed as a potential conflict of interest.

## Publisher’s Note

All claims expressed in this article are solely those of the authors and do not necessarily represent those of their affiliated organizations, or those of the publisher, the editors and the reviewers. Any product that may be evaluated in this article, or claim that may be made by its manufacturer, is not guaranteed or endorsed by the publisher.
